# Complications and Hospital Resource Utilization Associated with ECMO in COVID-19 ARDS with Acute Kidney Injury: Insights from United States National Inpatient Sample, 2020–2022

**DOI:** 10.3390/healthcare14203490

**Published:** 2026-10-17

**Authors:** Antony Arumairaj, Poojaben Dhorajiya, Dili Dhanani, Jyothika Venkataswamy Reddy, Abhishek Kumar Mariswamy Arun Kumar, Fatema Ali Asgar Tashrifwala, Vipulkumar Prajapati, Sireesha Palkamsetti, Dheera Tamvada, Digantkumar Patel, Divya Korpu, Jayesh Mittal

**Affiliations:** 1 Woodhull Medical Center, NYU Langone, Brooklyn, NY 11206, USA; 2 University of Kansas Hospital, Kansas City, KS 66160, USA; pdhorajiya@kumc.edu (P.D.);; 3 Chamarajanagar Institute of Medical Sciences, Chamarajanagar 571313, Karnataka, India; jyothikavreddy3004@gmail.com; 4 Department of Internal Medicine, Wright State University, Dayton, OH 45458, USA; 5 Stamford Hospital, Stamford, CT 06902, USA; fatema.tashrifwala12@gmail.com; 6 School of Medicine, Virginia Commonwealth University, Richmond, VA 23298, USA; vipulkumar.prajapati@vcuhealth.org; 7 Saint Agnes Medical Center, Fresno, CA 93720, USA; sireesha.palkamsetti@samc.com; 8 Golden Valley Health Centers, Merced, CA 95341, USA; dheeratamvada6@gmail.com; 9 Springfield Clinic, Springfield, IL 62703, USA; digantpatel4111987@gmail.com; 10 Mayo Clinic Health System, Eau Claire, WI 54703, USA; korpu.divya@mayo.edu; 11 Asante Rogue Regional Medical Center, Medford, OR 97504, USA

**Keywords:** COVID-19, acute respiratory distress syndrome, acute kidney injury, extracorporeal membrane oxygenation, mortality, mechanical ventilation, renal replacement therapy

## Abstract

**Highlights:**

**What are the main findings?**

- The use of extracorporeal membrane oxygenation (ECMO) was not associated with lower adjusted in-hospital mortality compared with invasive mechanical ventilation (IMV) alone in patients with COVID-19-associated acute respiratory distress syndrome (ARDS) and acute kidney injury (AKI).
- The patients who were on ECMO had a higher need for renal replacement therapy (RRT) and a longer hospital stay than those on IMV for COVID-19 ARDS with AKI.

**What are the implications of the main findings?**

- ECMO was used in a highly selected population of patients with COVID-19 ARDS and AKI, emphasizing the importance of careful patient selection.
- The increased requirement for RRT among patients on ECMO emphasizes the need for early nephrology involvement and renal-protective strategies in this high-risk population.

**Abstract:**

Background: Acute kidney injury (AKI) is a common complication of COVID-19-associated acute respiratory distress syndrome (ARDS), yet the impact of extracorporeal membrane oxygenation (ECMO) on outcomes in this high-risk population remains unclear. Methods: We performed a retrospective, survey-weighted study using the National Inpatient Sample (NIS) database from 2020–2022 to compare outcomes among adults hospitalized with COVID-19-associated ARDS and AKI managed with invasive mechanical ventilation (IMV) alone versus IMV and ECMO. The primary outcome was in-hospital mortality, while secondary outcomes included renal replacement therapy (RRT), complications, hospital length of stay (LOS), and healthcare utilization. Results: Among 198,395 hospitalizations with COVID-19 ARDS complicated by AKI, 4730 received IMV and ECMO. ECMO use was not associated with statistically significantly lower adjusted in-hospital mortality (adjusted odds ratio [aOR] 0.96, 95% CI 0.81–1.12). However, ECMO was associated with greater odds of RRT, intracranial hemorrhage, disseminated intravascular coagulation, cardiogenic shock, vasopressor use, tracheostomy, acute liver failure, deep vein thrombosis, ischemic stroke and ICU-acquired weakness. ECMO was also associated with longer LOS and higher hospitalization charges. Conclusions: Among hospitalizations with COVID-19-associated ARDS and AKI, ECMO was not associated with statistically significantly lower adjusted in-hospital mortality but was associated with markedly higher complication burden, longer hospitalization, and substantially greater healthcare resource utilization.

## 1. Introduction

Acute respiratory distress syndrome (ARDS) is a severe form of respiratory failure characterized by widespread alveolar damage, impaired gas exchange, and refractory hypoxemia, leading to profound oxygen deprivation to the vital organs [1]. ARDS, by causing renal ischemia due to reduced renal blood flow and increased venous pressure, can lead to acute kidney injury (AKI) in about 40% of patients [2]. AKI is often associated with increased mortality, prolonged intensive care unit (ICU) stay, and a higher healthcare resource burden [3,4]. COVID-19 is found to cause severe ARDS in 20–30% of hospitalized patients, often requiring ICU admission and invasive mechanical ventilation [5]. AKI has emerged as a frequent complication among these patients with rates exceeding 30–50% [6]. Multiple mechanisms likely contribute to renal injury in critically ill COVID-19 patients, including severe hypoxemia reducing renal perfusion, systemic inflammatory responses, high positive end-expiratory pressure (PEEP) caused by ventilation, reducing the cardiac output, and exposure to potentially nephrotoxic agents [7]. In some severe cases of COVID-19-associated ARDS, advanced respiratory support such as extracorporeal membrane oxygenation (ECMO) may be required when conventional invasive mechanical ventilation (IMV) fails to maintain adequate oxygenation. While ECMO can provide life-saving cardiopulmonary support, it has also been associated with a high incidence of AKI, likely due to systemic inflammation and complement activation from extracorporeal circuit exposure, hemolysis, hemodynamic instability, venous congestion, anticoagulation-related bleeding, and fluid overload [8]. Consequently, kidney injury is frequently observed in 60% of patients when ECMO is used to manage severe ARDS [9].

Despite growing evidence regarding ECMO use in severe COVID-19, comparative data remain limited in the specific high-risk population of patients with COVID-19-associated ARDS and concurrent AKI. Existing ECMO studies have largely evaluated broader populations with severe COVID-19 respiratory failure or ARDS, with limited comparative evidence focused specifically on patients with concomitant renal dysfunction. Therefore, we conducted a retrospective cohort study using the National Inpatient Sample from 2020 to 2022 to compare in-hospital mortality, renal replacement therapy, complications, length of stay, and hospitalization charges among adults with COVID-19-associated ARDS and AKI managed with IMV alone versus IMV plus ECMO.

## 2. Materials and Methods

### 2.1. Study Design and Data Source

We conducted a retrospective cohort study using the Healthcare Cost and Utilization Project (HCUP) National Inpatient Sample Database (NIS) from January 2020 to December 2022. The NIS is the largest inpatient database, with a survey design that collects discharge information from non-federal, non-rehabilitation, acute care, and short-term hospitals [10]. Its database is derived from billing data submitted by hospitals to statewide data organizations across the USA, covering more than 97% of the US population. Institutional Review Board (IRB) approval was not required because the HCUP NIS is a publicly available deidentified database and the analysis complied with the HCUP data use agreement, which contains no identifiable patient information.

### 2.2. Study Population

We included all patients admitted from January 2020 to December 2022 with a diagnosis of COVID-19-associated ARDS and AKI as defined by International Classification of Diseases 10th revision (ICD-10) codes. COVID-19 infection was identified using the ICD 10 code U07.1. Among these hospitalizations, acute respiratory distress syndrome was identified by using the ICD 10 code J80 and acute kidney injury was identified by using the ICD 10 codes N17.0, N17.1, N17.2, N17.8, and N17.9. The study population was divided into 2 groups. Group 1 included patients with COVID-19 ARDS with AKI managed with IMV alone, and group 2 included COVID-19 ARDS with AKI managed with IMV and ECMO.

### 2.3. Variables and Outcomes

IMV was identified using ICD-10-PCS procedure codes 5A1935Z, 5A1945Z and 5A1955Z. NIV before IMV was not an exclusion criterion; patients receiving non-invasive respiratory support were included if they also had an IMV procedure code during the hospitalization. ECMO exposure was identified using ICD-10-PCS procedure codes in any procedure field. Peripheral venoarterial (VA)-ECMO was identified by code 5A1522G, and peripheral venovenous (VV)-ECMO was identified by code 5A1522H. These codes were combined into a single ECMO exposure variable for the primary analysis. The primary outcome was in-hospital mortality. Secondary outcomes evaluated in the study included RRT, cardiogenic shock, vasopressor use, tracheostomy placement, acute liver failure, ischemic stroke, cardiac arrest, septic shock, intracranial bleeding, disseminated intravascular coagulation (DIC), acute pulmonary embolism (PE), acute deep vein thrombosis (DVT), ICU-acquired weakness, length of hospital stay, and total hospital charges.

Total hospital charges were analyzed as reported in the NIS using survey weights. Charges were not converted to costs using HCUP cost-to-charge ratio files and were not inflation-adjusted. The distribution of total hospital charges was additionally examined for extreme observations. The 99th percentile of total hospital charges was $2,469,918. Given the substantial right skew of the charge distribution, a log-transformed sensitivity analysis was also performed. We also evaluated discharge destinations and annual trends in ECMO utilization and mortality from 2020 to 2022. Variables used in this study included age, sex, race, median household income quartile, insurance, Charlson Comorbidity Index (derived from diagnosis fields and categorized as 0, 1, 2, and ≥3), hospital region, hospital location/teaching status, and hospital bed size.

### 2.4. Statistical Analysis

The patient information and data were analyzed using Stata/BE version 17.0 (StataCorp LLC, College Station, TX, USA). All analyses incorporated the NIS complex survey design, including discharge-level weights, strata, and hospital clusters, in accordance with HCUP recommendations. Survey-adjusted standard errors were calculated accounting for stratification and clustering to obtain nationally representative estimates, confidence intervals, and *p*-values. Continuous variables were reported as weighted means and compared using survey-weighted linear regression with adjusted Wald tests. Categorical variables were presented as weighted percentages and compared using the Rao–Scott corrected chi-square test. Survey-weighted univariable regression analyses were initially performed to estimate crude associations between ECMO exposure and each study outcome.

Survey-weighted multivariable logistic regression analyses estimated adjusted odds ratios for mortality, RRT, cardiogenic shock, vasopressor use, tracheostomy placement, acute liver failure, ischemic stroke, cardiac arrest, septic shock, intracranial bleeding, DIC, acute PE, acute DVT, and ICU-acquired weakness. Survey-weighted multivariable linear regression analyses estimated adjusted mean differences for LOS and total hospital charges. Because LOS and total hospital charges were right-skewed, sensitivity analyses were performed using log-transformed LOS and log-transformed total hospital charges. Variables included in the multivariable logistic and linear regression models were age, sex, race, median household income quartile, insurance status, Charlson Comorbidity Index, hospital region, hospital location/teaching status, hospital bed size and calendar year.

Given substantial baseline differences between the ECMO and IMV-only groups, propensity score matching (PSM) was performed as a sensitivity analysis. Propensity scores were estimated using logistic regression incorporating age, sex, race, median household income quartile, insurance, Charlson Comorbidity Index, hospital region, hospital location/teaching status, hospital bed size, hospital division, and calendar year. ECMO and non-ECMO hospitalizations were matched 1:1 using nearest-neighbor matching with replacement, a caliper of 0.2, and restriction to common support. Covariate balance before and after matching was assessed using standardized mean differences (SMDs), with an absolute SMD < 0.10 considered indicative of adequate balance. The PSM procedure did not incorporate the NIS complex survey design; primary analyses incorporated NIS discharge weights, strata, and hospital clusters, whereas matched analyses used propensity-score matching weights with robust standard errors.

Missing data were handled using complete-case analysis for each model. Multicollinearity among model covariates was assessed using variance inflation factors; the mean VIF was 2.31, indicating no evidence of problematic multicollinearity. Model sample sizes and event counts were recorded for each outcome model. Statistical significance was defined as a *p*-value < 0.05.

## 3. Results

### 3.1. Study Population

**Figure 1:**
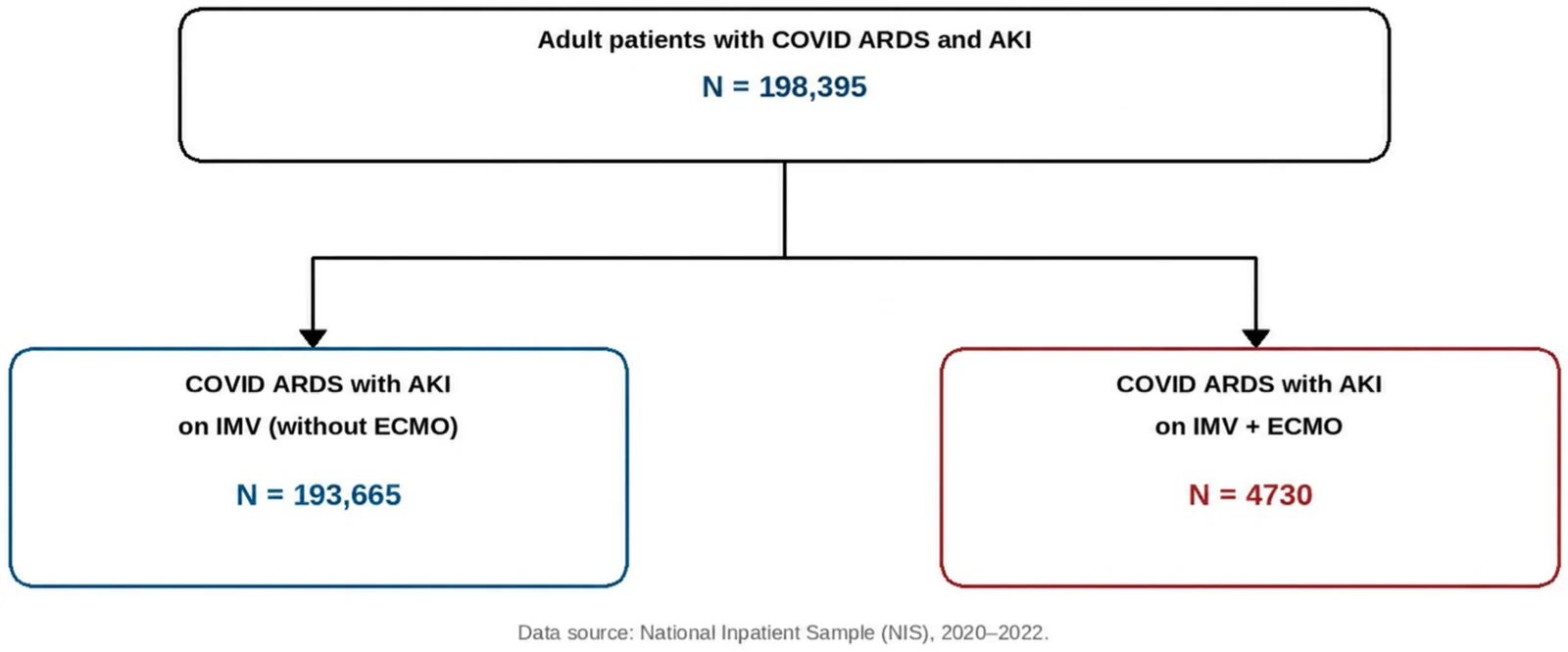
Flowchart of patient distribution in this study.

The final analytic cohort included 39,679 unweighted NIS discharge records, corresponding to a weighted national estimate of 198,395 adult hospitalizations for COVID-19-associated ARDS complicated by AKI requiring invasive mechanical ventilation. Of these, 38,733 unweighted records represented an estimated 193,665 hospitalizations managed with IMV without ECMO, while 946 unweighted records represented an estimated 4730 hospitalizations receiving IMV and ECMO (Figure 1).

### 3.2. Demographic and Hospital Characteristics

Patients hospitalized with ECMO were much younger than those handled with invasive mechanical ventilation alone (mean age 46.9 vs. 63.8 years, *p* < 0.001). Male patients accounted for a higher proportion of the ECMO group than the IMV-only group (71.7% vs. 63.1%), whereas females accounted for a lower proportion (28.3% vs. 36.9%; *p* < 0.001) (Table 1). Significant racial and ethnic disparities were found (*p* < 0.001). Caucasians were the largest racial group in both cohorts, although they accounted for a lower number of ECMO recipients (45.0% vs. 53.1%), whereas Hispanic patients were more common among ECMO recipients (25.2% vs. 20.1%). African Americans made up 19.5% of the ECMO cohort, compared to 18.0% of the IMV-only cohort. Socioeconomic parameters varied considerably (*p* = 0.008). Patients who had ECMO were less likely to be in the lowest national income quartile (29.4% vs. 34.1%), but more likely to be in the top income quartile (19.2% vs. 15.5%) (Table 1). Among patients undergoing ECMO, private insurance was the most common primary payer (59.3%), followed by Medicaid (26.9%). However, Medicare was the major payer among patients managed with invasive mechanical ventilation alone (52.6%), followed by private insurance (28.8%) (*p* < 0.001). Patients using ECMO had a reduced total comorbidity load, with fewer patients having a Charlson Comorbidity Index (CCI) ≥ 3 (18.8% vs. 40.1%) and a higher proportion having CCI ratings of 0 or 1 (*p* < 0.001). Hospitalizations were evenly distributed across geographic regions, with the Southern region accounting for the majority of admissions in both groups (40.1% vs. 37.5%; *p* = 0.078). ECMO was most commonly performed in urban teaching hospitals (95.9% vs. 78.2%) and major hospitals (83.6% vs. 51.6%) (*p* < 0.001) (Table 1). Substantial baseline imbalance was observed between the ECMO and IMV-only groups. Standardized differences were assessed as part of the propensity-score balance analysis; the mean standardized bias decreased from 40.1% before matching to 3.5% after matching (Supplementary Figure S1).

#### Clinical Outcomes

**Figure 2:**
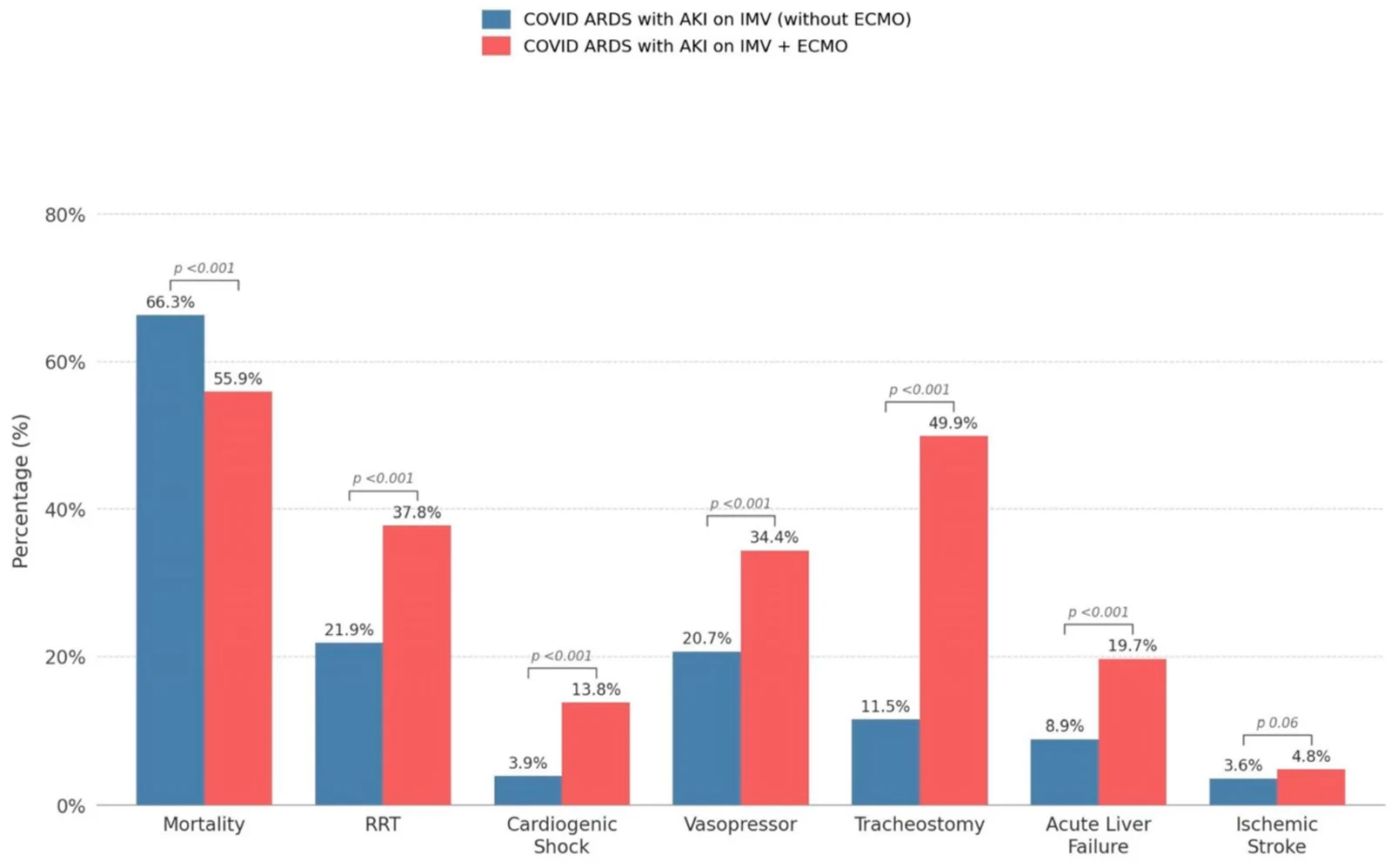
Clinical outcomes comparing Mortality, RRT, Cardiogenic shock, Vasopressor usage, Need for tracheostomy, Acute liver failure and Ischemic stroke in patients with COVID ARDS with AKI on IMV, with and without ECMO use.

Compared with patients managed with invasive mechanical ventilation alone, those receiving ECMO demonstrated a significantly greater complication burden and healthcare resource utilization. ECMO patients had significantly higher rates of renal replacement therapy (37.8% vs. 21.9%; aOR 1.52, 95% CI 1.29–1.79), cardiogenic shock (13.8% vs. 3.9%; aOR 3.33, 95% CI 2.62–4.23), vasopressor use (34.4% vs. 20.7%; aOR 1.63, 95% CI 1.29–2.06), tracheostomy (49.9% vs. 11.5%; aOR 4.56, 95% CI 3.87–5.37), and acute liver failure (19.7% vs. 8.9%; aOR 1.86, 95% CI 1.53–2.25) (all *p* < 0.001). Ischemic stroke was more frequent among ECMO recipients than among patients managed with IMV alone (4.8% vs. 3.6%; aOR 1.44, 95% CI 1.03–2.02; *p* = 0.03). The adjusted in-hospital mortality rate was not significantly different between the two groups (aOR 0.96, 95% CI 0.81–1.12; *p* = 0.61) (Figure 2).

**Figure 3:**
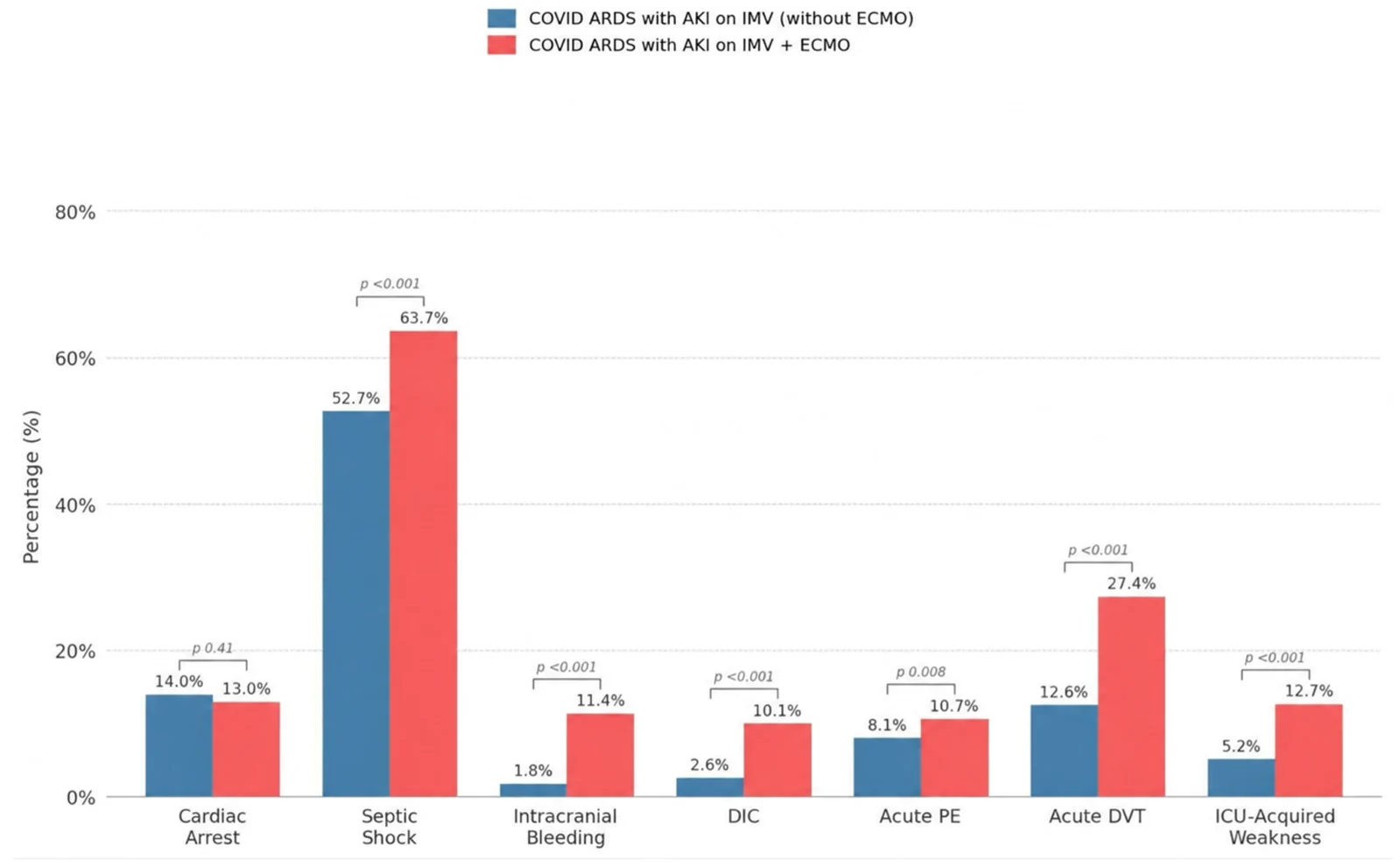
Clinical outcomes comparing Cardiac arrest, Septic shock, Intracranial bleeding, DIC, Acute PE, Acute DVT and ICU-acquired weakness in patients with COVID ARDS with AKI on IMV, with and without ECMO use.

ECMO patients also had significantly higher rates of septic shock (63.7% vs. 52.7%; aOR 1.30, 95% CI 1.11–1.53), intracranial bleeding (11.4% vs. 1.8%; adjusted odds ratio [aOR] 5.50, 95% CI 4.24–7.13), disseminated intravascular coagulation (DIC) (10.1% vs. 2.6%; aOR 3.59, 95% CI 2.71–4.76), acute deep venous thrombosis (27.4% vs. 12.6%; aOR 1.71, 95% CI 1.43–2.06) (all *p* < 0.001) and ICU-acquired weakness (12.7% vs. 5.2%; aOR 1.44, 95% CI 1.15–1.81, *p* = 0.001). However, the adjusted odds of cardiac arrest (aOR 0.84; 95% CI 0.67–1.05, *p* = 0.14) and acute pulmonary embolism (aOR 0.91, 95% CI 0.71–1.17, *p* = 0.47) were similar in the two groups (Figure 3). Propensity score matching substantially improved covariate balance, with mean standardized bias decreasing from 40.1% before matching to 3.5% after matching. All covariates achieved an absolute standardized mean difference <0.10 after matching. Covariate-specific absolute standardized mean differences before and after matching are shown in Supplementary Figure S1.

**Figure 4:**
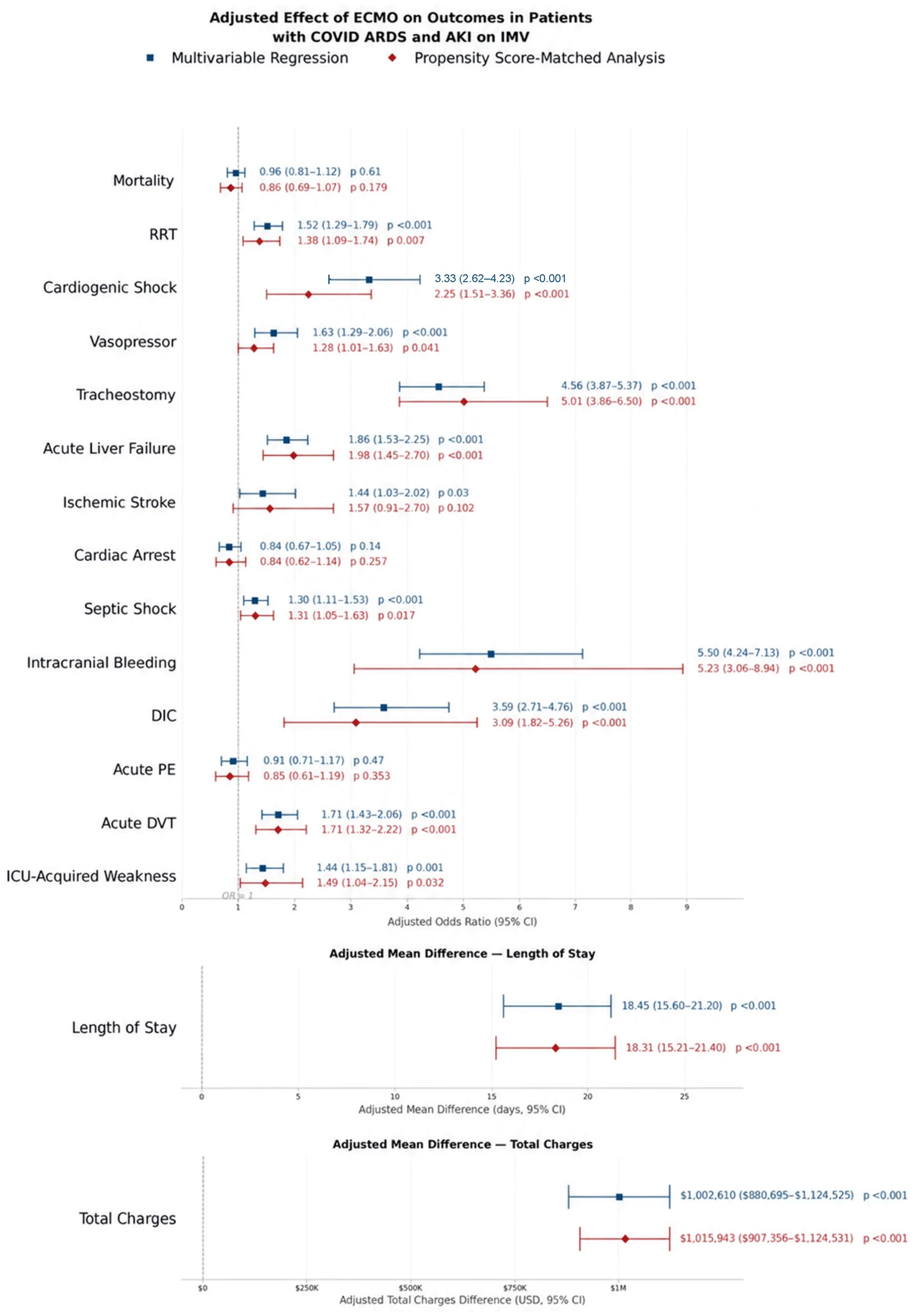
Forest plot showing multivariable regression and propensity score-matched (PSM) analyses of clinical outcomes among patients with COVID-19-associated ARDS and AKI.

Propensity score-matched analyses were generally consistent with the primary multivariable regression findings. ECMO use was not significantly associated with in-hospital mortality after matching (OR 0.86, 95% CI 0.69–1.07; *p* = 0.179). Associations with RRT, cardiogenic shock, vasopressor use, tracheostomy, acute liver failure, septic shock, intracranial bleeding, DIC, acute DVT, ICU-acquired weakness, longer length of stay, and higher total hospital charges remained statistically significant in the matched analysis (Figure 4; Supplementary Table S1). Ischemic stroke, cardiac arrest, and acute PE were not significantly associated with ECMO use after matching.

In sensitivity analyses stratified by clinically relevant subgroups, the adjusted association between ECMO use and in-hospital mortality varied across strata. ECMO use was associated with lower adjusted mortality among patients aged <50 years (aOR 0.73, 95% CI 0.58–0.92), those with CCI 0 (aOR 0.58, 95% CI 0.43–0.79), females (aOR 0.76, 95% CI 0.59–0.99), and hospitalizations in 2021 (aOR 0.73, 95% CI 0.59–0.91), whereas statistically significant associations were not observed in the remaining age, CCI, sex, and calendar-year strata. These subgroup analyses should be interpreted as exploratory (Supplementary Table S2).

**Figure 5:**
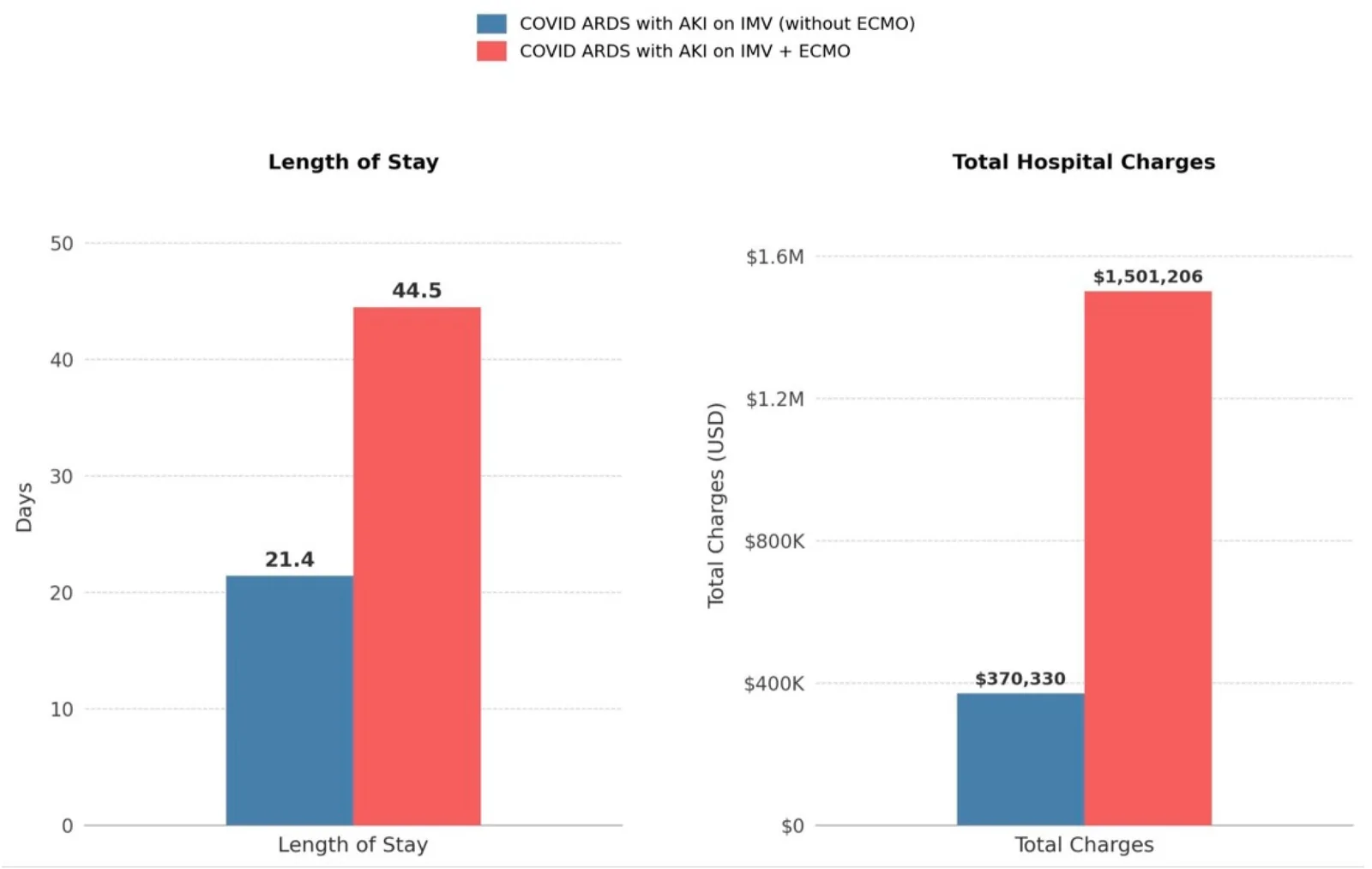
Length of hospital stay and total hospital charges for patients hospitalized with COVID ARDS and AKI on IMV, with and without ECMO use.

Patients receiving ECMO used much more healthcare resources. ECMO recipients had a considerably longer hospital stay than those managed with IMV alone (44.5 vs. 21.4 days), with an adjusted mean difference of 18.45 days (95% CI 15.6–21.2; *p* < 0.001). Similarly, total hospitalization charges were significantly greater in the ECMO cohort ($1,501,206 vs. $370,330), with an adjusted mean difference of $1,002,610 (95% CI $880,695–$1,124,525; *p* < 0.001) (Figure 5).

**Figure 6:**
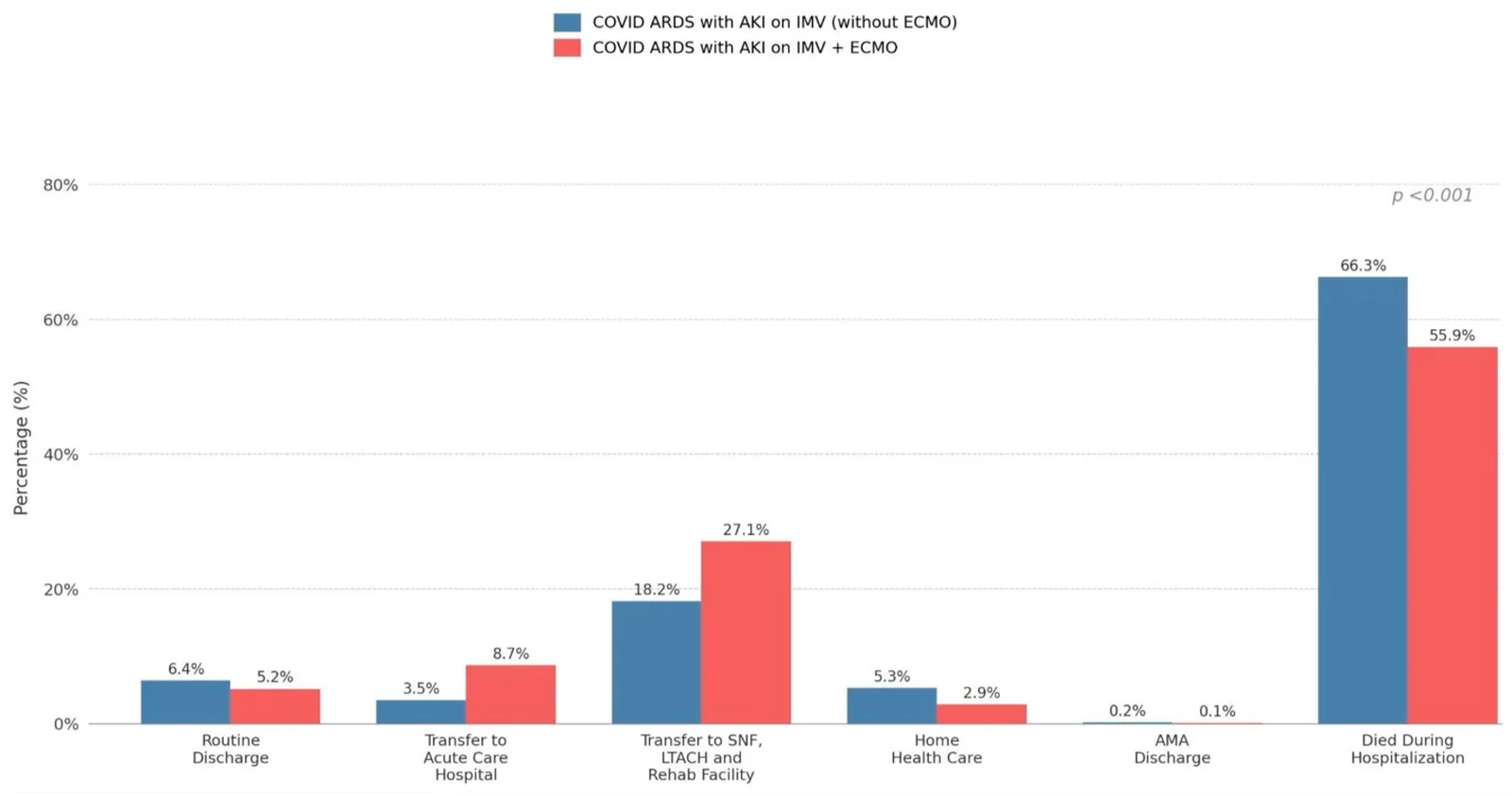
Discharge destinations for patients hospitalized with COVID ARDS and AKI on IMV, with and without ECMO use.

**Figure 7:**
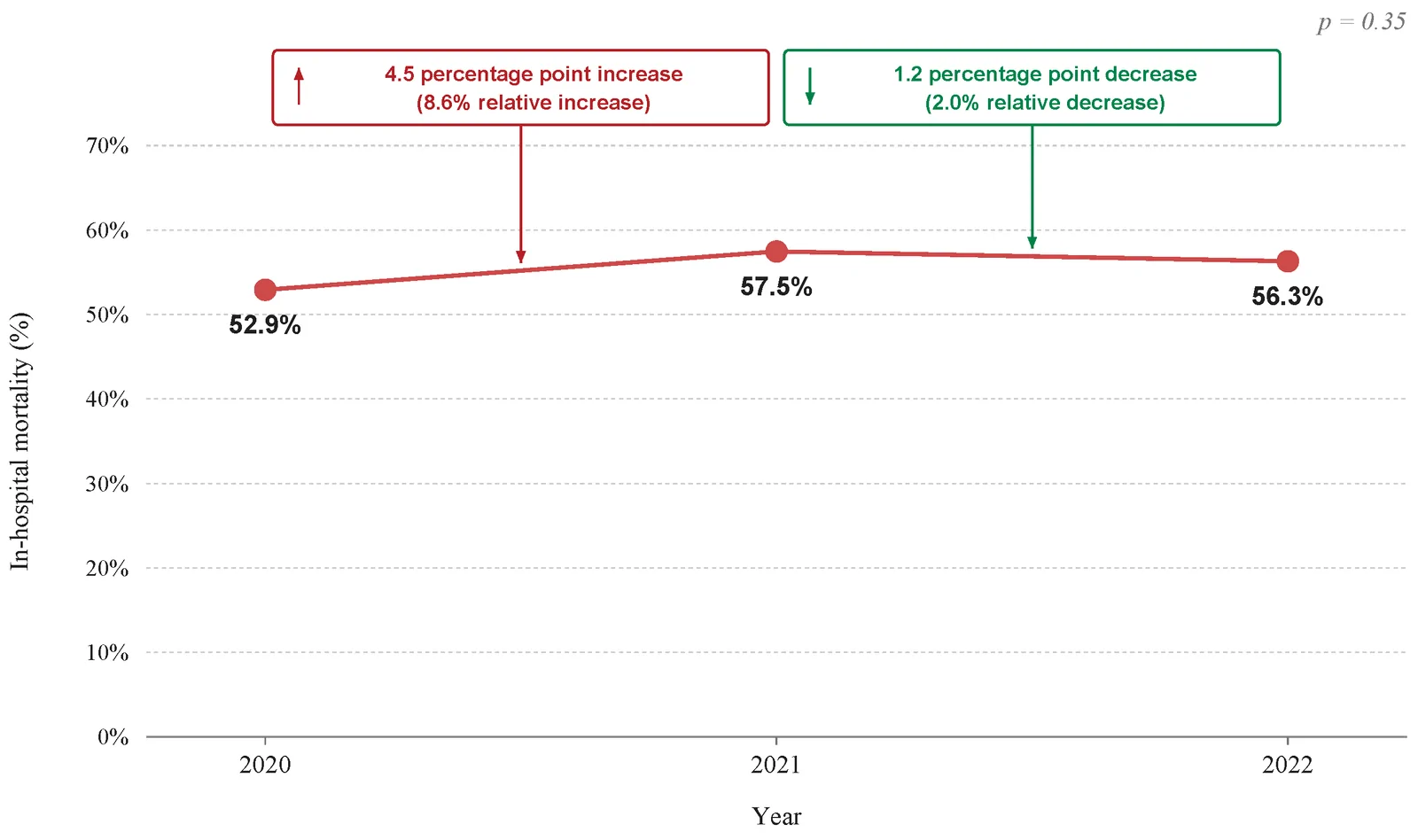
Mortality trends in patients with COVID ARDS with AKI on ECMO + IMV.

**Figure 8:**
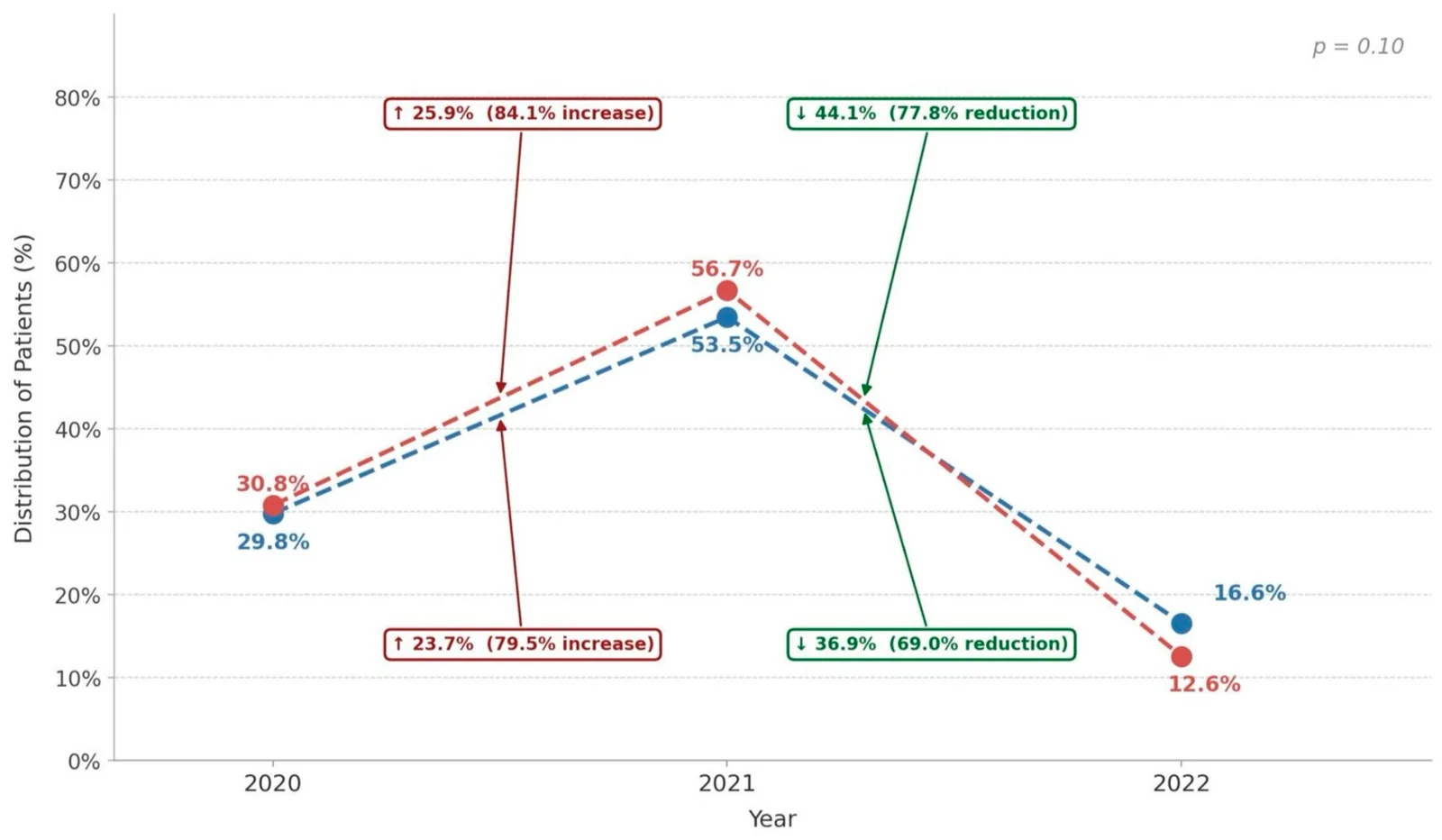
Distribution of patients with COVID ARDS and AKI on IMV, with and without ECMO use, in the years 2020–2022; blue dotted line—COVID ARDS with AKI on IMV; red dotted line—COVID ARDS with AKI on IMV + ECMO.

In-hospital death was the most prevalent discharge disposition in both groups, accounting for 55.9% of ECMO recipients and 66.3% of IMV-only hospitalizations. Transfer to skilled nursing facilities, long-term acute care hospitals, and rehabilitation facilities accounted for 27.1% of all ECMO recipients and 18.2% of IMV-only hospitalizations (*p* < 0.001) (Figure 6). These percentages were calculated among all hospitalizations. Among patients receiving ECMO, in-hospital mortality was 52.9% in 2020, 57.5% in 2021, and 56.3% in 2022, with no statistically significant linear trend across the study period (*p* = 0.35) (Figure 7). Among an estimated 4730 ECMO recipients during the study period, approximately 1455 (30.8%) occurred in 2020, 2680 (56.7%) in 2021, and 595 (12.6%) in 2022 (Figure 8).

## 4. Discussion

In this nationwide analysis of adults hospitalized with COVID-19-related ARDS complicated by AKI and requiring invasive mechanical ventilation, ECMO was used in a small, highly selected subgroup of patients. ECMO recipients were younger (46.9 vs. 63.8 years) and more often male (71.7% vs. 63.1%). ECMO recipients had fewer comorbidities with a Charlson Comorbidity Index ≥3 in only 18.8% compared with 40.1% in the IMV-only group. ECMO treatments were concentrated in urban teaching hospitals (95.9% vs. 78.2%) and large hospitals (83.6% vs. 51.6%). Although unadjusted mortality was lower among ECMO recipients (55.9% vs. 66.3%), ECMO was not independently associated with lower mortality after adjustment (aOR 0.96, 95% CI 0.81–1.12). In contrast, ECMO patients had a higher need for renal replacement therapy (37.8% vs. 21.9%; aOR 1.52, 95% CI 1.29–1.79) and markedly longer hospitalization (44.5 vs. 21.4 days). The lower crude mortality observed among ECMO recipients should be interpreted cautiously given substantial baseline differences between the groups, including age and comorbidity burden, as well as potential treatment-selection and referral effects [11,12,13,14,15,16,17]. These findings are consistent with our prior preliminary nationwide analyses of COVID-19-associated AKI and COVID-19 ARDS with AKI requiring ECMO; the present study expands on those observations using a larger 2020–2022 NIS cohort with multivariable, propensity score-matched, and subgroup sensitivity analyses [18,19].

The baseline characteristics observed in our ECMO cohort are consistent with the prior COVID-19 ECMO literature. The Extracorporeal Life Support Organization (ELSO) guidelines recommend ECMO as a rescue strategy for potentially reversible severe cardiopulmonary failure, with stricter selection criteria under resource constraints [11], and international COVID-19 ECMO registries consistently show that ECMO recipients tend to be younger, predominantly male, and treated at high-volume centers [12,14,20]. The demographic and hospital-level differences seen in our study, therefore, likely reflect referral patterns rather than treatment allocation independent of severity or expected reversibility [11,12,14,16,17,20].

Data from the early ELSO registry had shown acceptable survival among ECMO patients during the initial phase of the pandemic. However, subsequent analyses showed worsening outcomes as patient characteristics changed and center adoption evolved [12,13]. Patients on ECMO had a higher complication rate. Patients on ECMO also had higher mortality, as shown in the prospective EuroECMO-COVID study [21]. Nevertheless, comparative studies indicate that ECMO may reduce mortality in carefully chosen patients with profound refractory hypoxemia [14,15,16,17]. The absence of a statistically significant adjusted association between ECMO use and lower in-hospital mortality should not be interpreted as evidence of absence of a survival benefit. Residual confounding from unmeasured disease severity, treatment-selection and referral effects, timing of ECMO initiation, and the inability to account formally for ECMO as a time-dependent exposure may have influenced the observed association. Patients with COVID-19 ARDS and AKI represent a population with substantial multisystem illness and a high risk of adverse outcomes [1,2,6,22,23,24,25]. These clinical factors, together with the unmeasured severity and treatment-selection factors inherent to the NIS, limit interpretation of the observed mortality association [2,4,6,7,24,25]. Accordingly, the present findings should be interpreted as associative and do not establish either a survival benefit or absence of benefit from ECMO [12,13,14,15,16,17,20,21,23,24,25,26].

ECMO can result in hemodynamic instability, systemic inflammation, hemolysis, and coagulation abnormalities, leading to kidney injury [8,27,28]. There is an increase in the need for renal replacement therapy among patients treated with ECMO [8,26,27,28]. A meta-analysis of adult ECMO studies reported AKI in 62.8% of patients and severe AKI requiring RRT in 44.9%; AKI requiring RRT was strongly linked to increased mortality [26]. Because all patients in the analytic cohort had coded AKI, the higher RRT utilization observed among ECMO recipients may reflect greater severity of illness, more severe AKI, longer exposure time, or differences in clinical management rather than an ECMO-associated nephrotoxic effect. In addition, because the NIS lacks timing information for AKI onset, ECMO initiation, and RRT initiation, this association cannot establish that ECMO caused renal injury or RRT requirement.

The substantially longer hospital stay observed in the ECMO group also aligns with the prior literature and likely reflects the complexity of care needed for these patients. ECMO support in severe COVID-19 often involves prolonged cannulation, anticoagulation, treatment of bleeding and thrombotic complications, ventilator management, and extensive rehabilitation [11,21,29,30]. In EuroECMO-COVID, the median ECMO duration was 15 days, and complications occurred in most patients [21]. The longer length of stay and substantially higher total hospital charges observed among ECMO recipients are expected given the complexity and resource intensity of ECMO care.

Prior NIS-based analyses of COVID-19 hospitalizations have similarly demonstrated substantial resource utilization among high-risk comorbid populations, including patients with diabetes mellitus and hypertension [31,32]. More specifically, the substantially greater resource utilization observed among ECMO recipients is consistent with previous NIS-based studies demonstrating prolonged hospitalization and markedly higher hospital charges associated with ECMO use in mechanically ventilated patients with COVID-19 ARDS, patients with COVID-19 receiving VV-ECMO, and broader adult ECMO populations [33,34,35]. However, the longer LOS and higher hospital charges observed among ECMO recipients may also reflect greater illness severity, prolonged survival, extended mechanical ventilation, and greater opportunity to accumulate healthcare utilization. ELSO registry data and EuroECMO reports similarly show that ECMO-supported patients often require prolonged critical care, extended extracorporeal support, anticoagulation management, renal support, rehabilitation, and treatment of bleeding, thrombotic, and infectious complications [11,21,29,30]. The high concentration of ECMO care in urban teaching hospitals in our cohort further emphasizes that ECMO remains a highly specialized and resource-intensive therapy that depends on institutional expertise and multidisciplinary collaboration [11,21,29,30].

Beyond mortality, renal replacement therapy, and length of stay, ECMO use was associated with a substantially greater burden of adverse clinical events. Patients receiving ECMO had higher adjusted odds of intracranial bleeding, ischemic stroke, tracheostomy placement, disseminated intravascular coagulation, cardiogenic shock, vasopressor use, acute liver failure, septic shock, acute deep venous thrombosis, and ICU-acquired weakness. The higher rate of acute liver failure observed among ECMO recipients is consistent with prior NIS-based evidence showing that COVID-19-associated liver failure is linked to greater mortality, renal complications, ventilatory support, and hospital resource utilization [36]. The strongest associations were observed for intracranial bleeding and tracheostomy, which may reflect the combined effects of systemic anticoagulation, prolonged extracorporeal support, severe critical illness, extended mechanical ventilation, and greater opportunity for event detection among patients surviving long enough to receive ongoing intensive care. Many of these events, including bleeding, thrombosis, coagulopathy, renal replacement therapy, and tracheostomy, are recognized complications and downstream markers of ECMO-supported critical illness [8,21,26,27,28,29,30]. However, because the NIS is an administrative discharge-level database, temporal relationships between ECMO initiation and subsequent complications cannot be established. Therefore, these findings should be interpreted as associations rather than causal effects of ECMO. Although intracranial bleeding may plausibly be related to systemic anticoagulation and ECMO-associated hemostatic abnormalities, the higher occurrence of DVT may reflect multiple overlapping factors, including the thromboinflammatory state associated with severe COVID-19, prolonged immobilization and critical illness, as well as ECMO-related factors. The strong association between ECMO use and tracheostomy should be interpreted as a time-dependent and survivor-related outcome, as ECMO recipients frequently require prolonged mechanical ventilation and must survive long enough for tracheostomy to be performed. In contrast, the adjusted risks of cardiac arrest and acute pulmonary embolism were not significantly different between the groups. These findings emphasize that ECMO support in patients with COVID-19-associated ARDS and AKI is accompanied by considerable bleeding, thrombotic, cardiovascular and neuromuscular morbidity, further supporting careful patient selection and close multidisciplinary monitoring.

Our findings demonstrate that ECMO was used in a highly selected population of patients with COVID-19-associated ARDS complicated by AKI, consistent with its established role as a rescue therapy in appropriately selected patients [11,15,16,17]. The notably higher renal replacement therapy requirements in this population underscore the need for early nephrology consultation, close renal monitoring, and renal-protective supportive care [2,8,24,26,27]. Additionally, the concentration of ECMO use in large urban teaching hospitals suggests that access to this treatment remains largely limited to specialized tertiary centers with the necessary expertise, infrastructure, and multidisciplinary resources [11,15,16,17,21,29,30]. ECMO was also associated with substantially greater hospital charges, with an adjusted mean difference of more than $1.0 million compared with IMV alone, highlighting the major economic burden of ECMO use in this high-risk population.

### Limitations

Our study has several limitations. The NIS is a discharge-level administrative database rather than a patient-level longitudinal registry, limiting the ability to identify repeat admissions or assess long-term outcomes [10,37]. Administrative databases are also subject to coding inaccuracies and lack granular physiologic and clinical data relevant to critical care outcomes research [10,37]. Additionally, complications were identified using administrative diagnosis and procedure codes and may therefore be subject to misclassification and differential coding intensity between ECMO centers and non-ECMO hospitals; the NIS also does not permit determination of whether these complications preceded or followed ECMO initiation. ARDS was identified using ICD-10-CM code J80; therefore, misclassification is possible because the NIS does not include clinical criteria required to verify ARDS diagnosis, including PaO2/FiO2 ratio, PEEP requirements, chest imaging findings, timing of respiratory failure, or clinician-adjudicated ARDS diagnosis. AKI was identified using ICD-10-CM codes; however, the NIS does not provide serum creatinine values, urine output, Kidney Disease: Improving Global Outcomes (KDIGO) stage, timing of AKI onset, ECMO initiation, or RRT initiation. Therefore, we could not determine AKI severity or whether AKI occurred before ECMO, after ECMO initiation, or during the same hospitalization. Therefore, AKI severity and temporal relationships between ECMO and renal outcomes could not be assessed. The database also lacks key severity and treatment variables such as ventilator settings, SOFA score, lactate level, vasopressor dose, prone positioning, inflammatory markers, nephrotoxic exposures, AKI staging, dialysis modality, vaccination status, viral variant, center-level ECMO volume, and ECMO-related details such as timing of initiation, duration of support, and cannulation strategy [10].

Although multivariable regression and propensity score-matched analyses were performed to reduce confounding from measured patient and hospital characteristics, the substantial baseline differences between the ECMO and IMV-only groups indicate strong treatment-selection and referral effects; therefore, residual confounding, referral bias, indication bias, and survivor bias remain possible [10,37,38]. In particular, the mortality comparison may be affected by survivor bias because patients must survive long enough to undergo evaluation, referral, and initiation of ECMO, while the NIS lacks the temporal information necessary to formally account for this potential immortal-time effect. Outcomes requiring prolonged survival, including tracheostomy, rehabilitation transfer, and ICU-acquired weakness, may be particularly susceptible to survivor bias and competing risk from in-hospital death; therefore, crude comparisons of these outcomes should be interpreted cautiously. ECMO candidacy is influenced by clinical severity, physiologic parameters, timing of respiratory failure, center expertise, and resource availability, which are not fully captured in the NIS [10,37]. ECMO recipients also tended to be younger, had fewer comorbidities, and were treated predominantly at large tertiary referral centers [11,12,13,14,15,37,38]. Accordingly, the observed associations should be interpreted as associative rather than causal [10,37,38]. Secondary outcome analyses were considered exploratory. Given the number of secondary outcomes evaluated and the absence of formal adjustment for multiple comparisons, the possibility of type I error should be considered when interpreting these findings.

## 5. Conclusions

In patients with COVID-19-associated ARDS complicated with AKI requiring mechanical ventilation, ECMO was used in a highly selected subset of the population who were younger, had fewer comorbidities, and received care at large urban teaching hospitals. The crude mortality was lower in ECMO recipients. However, ECMO was not associated with statistically significantly lower in-hospital mortality after adjustment in this administrative database analysis. ECMO use was associated with a greater need for RRT and substantially longer hospitalization and was observed predominantly among carefully selected patients treated at experienced centers. These findings underscore the significant burden of multisystem organ dysfunction among patients with COVID-19 ARDS complicated by AKI and highlight the complexity of identifying patients most likely to benefit from ECMO support. Future research should refine ECMO selection criteria, evaluate strategies to mitigate renal complications, and assess long-term renal and survival outcomes in this high-risk population.

## Figures and Tables

**Table 1:** Demographic and Clinical Characteristics of Hospitalized COVID-19 Patients with ARDS and AKI on Invasive Mechanical Ventilation, stratified by ECMO use.

Variable	COVID ARDS with AKI on IMV (*n* = 193,665)	COVID ARDS with AKI on IMV + ECMO (*n* = 4730)	*p*-Value
Age, mean (years)	63.8	46.9	<0.001
Sex			<0.001
Male	63.1%	71.7%	
Female	36.9%	28.3%	
Race			<0.001
Caucasian	53.1%	45.0%	
African American	18.0%	19.5%	
Hispanic	20.1%	25.2%	
Asian/Pacific Islander	3.6%	4.2%	
Native American	1.3%	2.0%	
Other	3.8%	4.0%	
National Income Quartile			0.008
1–$38,999	34.1%	29.4%	
39,000–47,999	26.8%	26.6%	
48,000–62,999	23.6%	24.8%	
>$63,000	15.5%	19.2%	
Insurance			<0.001
Medicare	52.6%	9.6%	
Medicaid	15.0%	26.9%	
Private	28.8%	59.3%	
Uninsured	3.6%	4.1%	
Charlson Comorbidity Index			<0.001
0	17.9%	30.2%	
1	23.0%	31.7%	
2	18.9%	19.2%	
≥3	40.1%	18.8%	
Hospital Region			0.078
Northeast	17.4%	16.1%	
Mid-West	20.7%	26.5%	
South	40.1%	37.5%	
West	21.8%	19.9%	
Hospital Location & Teaching Status			<0.001
Rural	6.3%	1.1%	
Urban, Non-Teaching	15.5%	3.1%	
Urban, Teaching	78.2%	95.9%	
Hospital Bed Size			<0.001
Small	20.4%	5.4%	
Medium	27.9%	11.0%	
Large	51.6%	83.6%	

NIS = National Inpatient Sample; IMV = Invasive Mechanical Ventilation; ECMO = Extracorporeal Membrane Oxygenation; ARDS = Acute Respiratory Distress Syndrome; AKI = Acute Kidney Injury.

## Data Availability

The data analyzed in this study are derived from the Healthcare Cost and Utilization Project (HCUP) National Inpatient Sample (NIS), maintained by the Agency for Healthcare Research and Quality (AHRQ). The dataset is publicly available and can be accessed at https://www.hcup-us.ahrq.gov/nisoverview.jsp (accessed on 15 March 2026).

## Supplementary Materials

The following supporting information can be downloaded at https://www.mdpi.com/article/10.3390/healthcare14203490/s1, Table S1: Propensity Score Matched Outcomes Comparing ECMO vs No ECMO Matched analysis of adult patients with COVID-19 ARDS, AKI, and invasive mechanical ventilation; Table S2: Subgroup Sensitivity Analysis for In-Hospital Mortality; Figure S1: Covariate Balance Before and After Propensity Score Matching.

## Abbreviations

AKI	Acute Kidney Injury
ARDS	Acute Respiratory Distress Syndrome
COVID-19	Coronavirus Disease 2019
ECMO	Extracorporeal Membrane Oxygenation
HCUP	Healthcare Cost and Utilization Project
ICU	Intensive Care Unit
IMV	Invasive Mechanical Ventilation
LOS	Length of Stay
NIS	National Inpatient Sample
PEEP	Positive End-Expiratory Pressure
RRT	Renal Replacement Therapy
