# Supplementary material for: Complications and Hospital Resource Utilization Associated with ECMO in COVID-19 ARDS with Acute Kidney Injury: Insights from United States National Inpatient Sample, 2020–2022
Source: Healthcare (Basel). 2026 Oct 17;14(20):3490. doi: 10.3390/healthcare14203490 (PMC13704019; doi:10.3390/healthcare14203490)
Supplement: Supplementary file 1 [file healthcare-14-03490-s001.zip › healthcare-4539194-supplementary.pdf]

Supplementary Materials

Supplementary Table S1. Propensity Score Matched Outcomes Comparing ECMO vs No ECMO
Matched analysis of adult patients with COVID-19 ARDS, AKI, and invasive mechanical ventilation.

Outcome	Matched N	PSM adjusted estimate	95% CI	P value
Mortality	1,503	0.86	0.69-1.07	0.179
RRT	1,503	1.38	1.09-1.74	0.007
Cardiogenic shock	1,399	2.25	1.51-3.36	<0.001
Vasopressor use	1,503	1.28	1.01-1.63	0.041
Tracheostomy	1,503	5.01	3.86-6.50	<0.001
Acute liver failure	1,503	1.98	1.45-2.70	<0.001
Ischemic stroke	1,421	1.57	0.91-2.70	0.102
Cardiac arrest	1,503	0.84	0.62-1.14	0.257
Septic shock	1,503	1.31	1.05-1.63	0.017
Intracranial bleeding	1,460	5.23	3.06-8.94	<0.001
DIC	1,482	3.09	1.82-5.26	<0.001
Acute PE	1,503	0.85	0.61-1.19	0.353
Acute DVT	1,503	1.71	1.32-2.22	<0.001
ICU-acquired weakness	1,503	1.49	1.04-2.15	0.032
Length of stay, days	1,501	+18.31	15.21-21.40	<0.001
Total charges, USD	1,481	+1,015,943	907,356-1,124,531	<0.001

Note: Values are extracted from the Stata analysis log. Binary outcomes are propensity-score-matched adjusted odds ratios; length of stay and total charges are adjusted mean differences. Models used robust standard errors and adjusted for demographics, Charlson index, hospital characteristics, division, and year. Post-match balance improved: mean standardized bias 40.1 to 3.5; median standardized bias 17.2 to 4.0; pseudo-R2 0.231 to 0.004; omnibus balance p=0.648.

Supplementary Table S2. Subgroup Sensitivity Analysis for In-Hospital Mortality

Subgroup	Stratum	Adjusted OR	95% CI	P value
Age	<50	0.73	0.58-0.92	0.007
Age	50-64	0.80	0.64-1.01	0.059
Age	>=65	0.71	0.36-1.41	0.330
Sex	Male	0.82	0.67-1.00	0.053
Sex	Female	0.76	0.59-0.99	0.045
Charlson Comorbidity Index	0	0.58	0.43-0.79	<0.001
Charlson Comorbidity Index	1	0.79	0.60-1.03	0.077
Charlson Comorbidity Index	2	1.12	0.79-1.59	0.528
Charlson Comorbidity Index	>=3	1.00	0.71-1.43	0.983
Calendar year	2020	0.92	0.69-1.22	0.547
Calendar year	2021	0.73	0.59-0.91	0.005
Calendar year	2022	0.88	0.58-1.32	0.529

Note: Adjusted odds ratios compare ECMO versus no ECMO within each subgroup. Estimates are survey-weighted logistic regression results from the analysis log. Age-stratified models adjusted for sex, race, income quartile, insurance, Charlson index, hospital characteristics, and year. Sex-stratified models adjusted for age, race, income quartile, insurance, Charlson index, hospital characteristics, and year. CCI-stratified models adjusted for age, sex, race, income quartile, insurance, hospital characteristics, and year. Year-stratified models adjusted for age, sex, race, income quartile, insurance, Charlson index, and hospital characteristics.

Supplementary Figure S1. Covariate Balance Before and After Propensity Score Matching

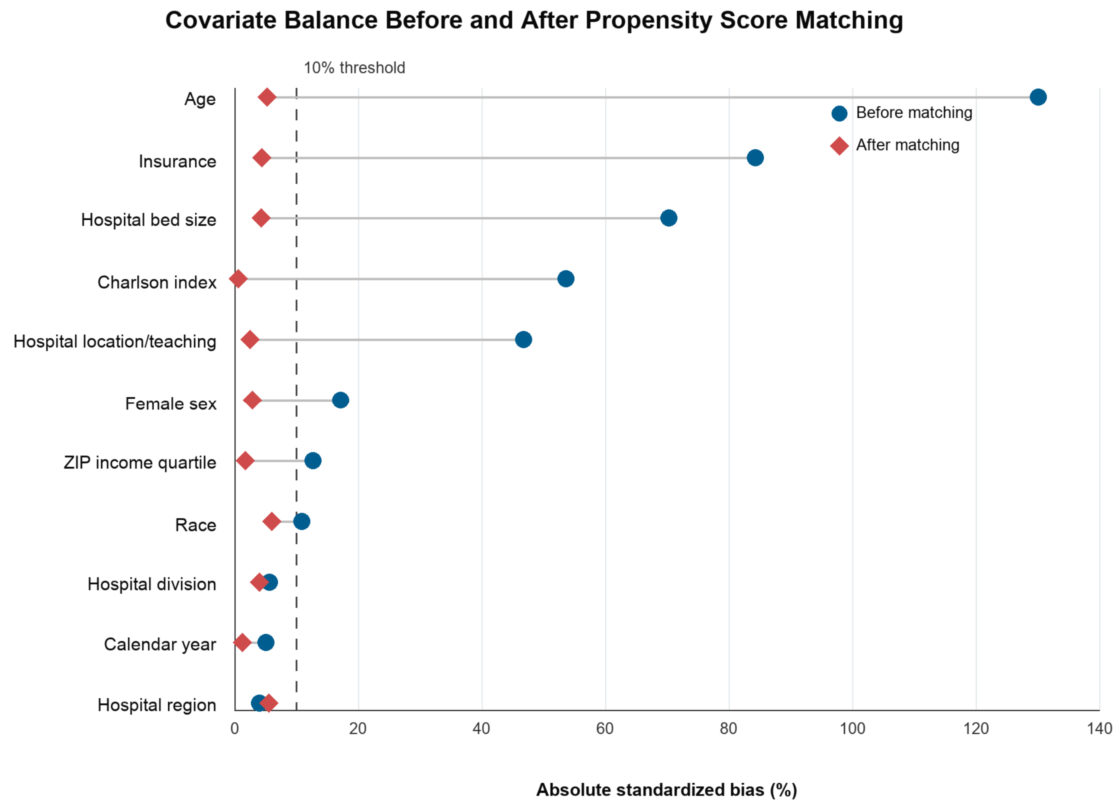

Note: The figure uses the standardized-bias diagnostics available in the Stata log for the covariates included in the propensity score model. Points show absolute standardized bias before and after matching. The log reports aggregate balance improvement from mean standardized bias 40.1% to 3.5%, median standardized bias 17.2% to 4.0%, pseudo-R2 0.231 to 0.004, and omnibus balance P=0.648 after matching. Variable-level values are not category-expanded beyond what is provided by the log.
